# The arable ecosystem as battleground for emergence of new human pathogens

**DOI:** 10.3389/fmicb.2014.00104

**Published:** 2014-03-20

**Authors:** Leonard S. van Overbeek, Joop van Doorn, Jan H. Wichers, Aart van Amerongen, Herman J. W. van Roermund, Peter T. J. Willemsen

**Affiliations:** ^1^Plant Research International, Wageningen University and Research CentreWageningen, Netherlands; ^2^Applied Plant Research, Wageningen University and Research CentreLisse, Netherlands; ^3^Food and Biobased Research, Wageningen University and Research CentreWageningen, Netherlands; ^4^Central Veterinary Institute, Wageningen University and Research CentreLelystad, Netherlands

**Keywords:** EHEC, emerging pathogens, phytonoses, gene transfer, rhizosphere, *Enterobacteriaceae*

## Abstract

Disease incidences related to *Escherichia coli* and *Salmonella enterica* infections by consumption of (fresh) vegetables, sprouts, and occasionally fruits made clear that these pathogens are not only transmitted to humans via the “classical” routes of meat, eggs, and dairy products, but also can be transmitted to humans via plants or products derived from plants. Nowadays, it is of major concern that these human pathogens, especially the ones belonging to the taxonomical family of *Enterobacteriaceae*, become adapted to environmental habitats without losing their virulence to humans. Adaptation to the plant environment would lead to longer persistence in plants, increasing their chances on transmission to humans via consumption of plant-derived food. One of the mechanisms of adaptation to the plant environment in human pathogens, proposed in this paper, is horizontal transfer of genes from different microbial communities present in the arable ecosystem, like the ones originating from soil, animal digestive track systems (manure), water and plants themselves. Genes that would confer better adaptation to the phytosphere might be genes involved in plant colonization, stress resistance and nutrient acquisition and utilization. Because human pathogenic enterics often were prone to genetic exchanges via phages and conjugative plasmids, it was postulated that these genetic elements may be hold key responsible for horizontal gene transfers between human pathogens and indigenous microbes in agroproduction systems. In analogy to zoonosis, we coin the term phytonosis for a human pathogen that is transmitted via plants and not exclusively via animals.****

## INTRODUCTION

### THE PLANT ENVIRONMENT AS A HABITAT FOR HUMAN PATHOGENS

Agricultural plants have become a source for human pathogens, especially the emerging ones belonging to the group of Shiga toxin-producing *Escherichia coli* (STEC) strains (Feng and Reddy, 2013). The threat of human pathogens in freshly consumable products of plant origin became apparent during the outbreak caused by *Escherichia coli* O104:H4 in Germany and France in 2011, where almost 4000 persons became infected leading to 54 casualties and over 900 incidences of hemolytic uremic syndrome (HUS; Karch et al., 2011; Beutin and Martin, 2012). The most likely transmission route of the pathogen to consumable products was remarkable as the source was fenugreek seeds that were transported from Egypt to Rotterdam harbor, the Netherlands, approximately 17 months before appearance of the first disease incidences in Hamburg and surrounding area (Karch et al., 2011). Although the pathogen was neither found in Fenugreek sprouts, nor in the seeds themselves, epidemiological facts revealed that the pathogen must have been associated with seeds over a relatively long period of time. The questions rise on how a human pathogen can persist as a viable entity in a hostile environment for such a long period and why similar observations had not been made before. Must this be considered as the first incidence of a disease outbreak caused by a human pathogenic bacterium that was adapted to the plant environment?

To address this question, it must be referred to the nature of the causative agent that is atypical for pathogenic *Escherichia coli* strains commonly occurring in Europe and the USA (Scheutz et al., 2011; Beutin and Martin, 2012). The *Escherichia coli* O104: H4 outbreak strain was an entero-aggregative *Escherichia coli* strain that does not have animals, which is often the case for other *Escherichia coli* O type strains, but instead only humans as major reservoir (Wieler et al., 2011). Outbreaks caused by this type of pathogen are rare in Western societies, whereas those caused by *Escherichia coli* O157:H7 and *Salmonella enterica* are more common (**Table 1**). This indicates that particular features in these human pathogens already exist, extending their life-time in the plant environment. The question is whether these features were intrinsic to particular subsets of human pathogens or were recently gained, e.g., via horizontal gene transfer. The strain causing the outbreak in the Hamburg area must be considered as a highly virulent pathogen and it must have acquired its virulence and antibiotic resistance (extended spectrum beta-lactamase, ESBL) traits via horizontal gene transfer events like transduction (phage infection) and conjugation (plasmid transfer; Mellmann et al., 2011; Muniesa et al., 2012). Acquisition of new virulence traits is one aspect in the evolution of a new pathogen, but selection pressure is another, and the outbreak strain must have been evolved by increasing its virulence in humans side-by-side with improvement of its ecological competence in plants. The threat of this development is the emergence of new types of highly virulent human bacterial pathogens that are fully adapted to life near, or may be inside agricultural plants.

**Table 1:** Examples of large disease outbreaks resulting from contamination of vegetables and sprouts with human pathogenic bacteria.

Location	Pathogen	Food source	Reference
East Anglia (UK)	*E. coli* O157:H7	Potato tubers	Morgan et al. (1988)
Osaka (Japan)	*E. coli* O157:H7	Radish sprouts	Michino et al. (1999)
Connecticut (USA)	*E. coli* O157:H7	Mesclun lettuce	Hilborn et al. (1999)
Western USA, British Columbia (Canada)	*E. coli* O157:H7	Unpasteurized apple juice	Cody et al. (1999)
California (USA)	*E. coli* O157:H7, *S. enterica*	Alfalfa and clover sprouts	Mohle-Boetani et al. (2001)
Michigan (USA)	*E. coli* O157:H7	Alfalfa sprouts	Breuer et al. (2001)
Multistate outbreak USA	*E. coli* O157:H7	Packaged spinach	Wendel et al. (2009)
Hamburg (Germany)	*E. coli* O104:H4	Fenugreek sprouts	Bielaszewska et al. (2011)

### BACTERIA ASSOCIATED WITH MULTIPLE HABITATS

The agricultural plant environment is an environment where microbial communities of at least four different ecosystems may come together, namely those from soil, plants, farm animal digestive track systems (manure), and fresh water sources (irrigation). Agricultural plant production thus must be considered as a cross road of communities originating from these different sources. The microbial community compositions in these systems substantially differ from each other, but representatives of the different communities may, at least temporarily, coexist with micro-organisms typically associated with plants. However, much is still unknown about the microbial composition and functioning of these microbes in the different environments. Some remarkable correspondence in the taxonomy of human disease-causing bacteria can be found with typical soil/rhizosphere bacterial species. Clearest examples are the so called cross-domain pathogens belonging to the genera of *Burkhoderia* (e.g., *Burkhoderia cepacia*) and *Pseudomonas* (e.g., *Pseudomonas aeruginosa*; Barak and Schroeder, 2012; Kumar et al., 2013). These are opportunistic pathogens in humans and are commonly found in plant and soil habitats. Distinction of pathogenic from non-pathogenic types (of which some can be excellent plant growth promoters) of both genera is often difficult to accomplish. *Pseudomonas fluorescens* is a bacterial species that is commonly found near plant roots, however, representatives of this species were also shown to be associated with patients suffering from Crohn’s disease (Wei et al., 2002; Eckburg and Relman, 2007). A representative of *Pseudomonas veronii*, another typical rhizosphere-borne bacterial species was found to be associated with human intestinal inflammatory pseudotumour formation (Cheuk et al., 2000). Typical plant pathogens belonging to the genus of *Pantoea (Pantoea ananatis* and *P. agglomerans*) and *Erwinia* (*Erwinia tasmaniensis*) were also demonstrated to cause diseases in humans (Cruz et al., 2007; Shin et al., 2008; Coutinho and Venter, 2009). Taxonomical commonalities exist between human pathogens and species known to thrive in soil habitats. Recently, a new taxa belonging to a hitherto uncultured group of bacteria named *Verrucomicrobia* subdivision 1 was shown to be competent in the rhizospheres of different plant species (Nunes da Rocha et al., 2011), whereas a representative of the same group (*Akkermansia muciniphila*) had been found as a commensal species living in the intestinal track system of humans (Derrien et al., 2004), where it deemed to play a role in degradation of mucus and health support of humans. This illustrates that the taxonomical diversity of cross domain species are high and up to date still rather unexplored. Important proposition in this paper is that the chance on genetic exchanges via horizontal transfer will be highest between cross-domain species commonly occurring in different ecosystems and coming into physical contact with each other near or within plants. Human pathogens may acquire genes from plant-associated bacteria (Szmolka and Nagy, 2013), leading to new phenotypes of increased persistence in plants or their propagative materials, higher resistance to stresses or broader spectra in acquisition or utilization of available nutrients. These traits will make human pathogens fitter for survival in the plant environment.

Especially, the rhizosphere and phyllosphere of plants must be considered as hot spots of microbial life because of the nutrients leaking from growing roots and leaves. Plant surfaces are important reservoirs for (enteric) opportunistic human pathogens (Berg et al., 2005; Holden et al., 2009). *Escherichia coli* belong to the *Enterobacteriaceae*, and species belonging to this taxonomical class can be found frequently as mutualistic, commensal, or plant pathogenic species in plants. Species belonging to the genera of *Enterobacter, Serratia, Klebsiella, Erwinia, Pantoea, *and *Pectobacterium* are typical plant-associated bacteria and if gene transfer events with plant-invasive human pathogens, like ones belonging to *Escherichia coli* and *S. enterica*, will occur then expectedly it will be highest with these species. Prophages and plasmids in plant-associated *Enterobacteriaceae* are extrachromosomal gene pools that, thus far, remained inaccessible for human pathogens. On the other hand harmless bacteria living as commensals in plant environments may pick up mobile elements from plant-invasive human pathogenic species to become human pathogens (Szmolka and Nagy, 2013). The crucial difference between a relatively harmless bacterium and a dangerous human pathogen can rely on presence or absence of a single mobile genetic element (Frost et al., 2005). It, thus, might be possible that harmless bacterial species living as commensals in plants acquire traits making them virulent to humans although these type of incidences has not been reported to occur in plants to date.

**FIGURE 1:**
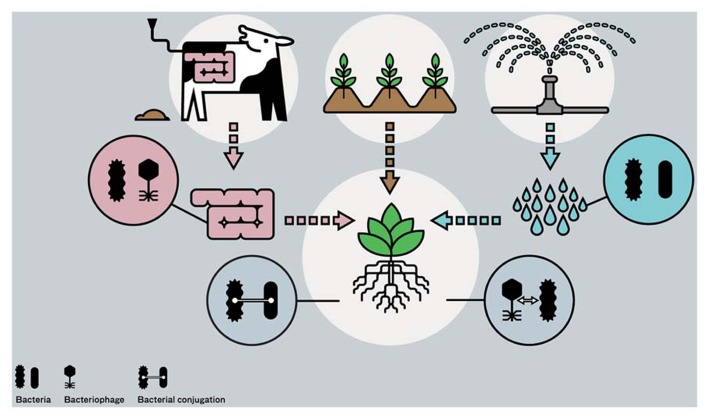
**Bacterial communities from different habitats, i.e., from cattle intestinal track system via manure, surface water via irrigation, and from soil and plants, all coming together at plant growth in arable production systems.** Four magnifications in the figure depict origin of different bacterial groups and their phages, and the possible occurrences of gene transfer near or inside plants, either via conjugation or via transduction.

Practices common in agriculture make it possible to bring gene pools from different ecosystems together and making these accessible for commensal and human pathogenic species, leading to acquisition of new traits and possibly to emergence of new human pathogens (**Figure 1**). Phytonoses (singular phytonosis), occurring from human infecting agents transmitted by plants (van der Riet, 1997), in analogy to zoonoses, which are diseases transmitted by animals, can be coined as a new term for the group of diseases caused by human pathogens, viz. *Escherichia coli* and *S. enterica*, that are transmitted via consumption of fresh produce.

## THE ARABLE PLANT ENVIRONMENT AS CROSS ROAD OF DIFFERENT ECOSYSTEMS

### THE ANIMAL DIGESTIVE TRACK SYSTEM AS MAJOR RESERVOIR FOR *Escherichia* coli O157:H7 AND OTHER HUMAN PATHOGENIC ENTERICS

Shiga toxin-producing *Escherichia coli* serotypes, the most commonly found human pathogens in plant-derived products, pose serious threats to human health. The question often raised in different studies is why these strains, and especially the ones of *Escherichia coli* O157:H7, are so often associated with plants (**Table 1**). *Escherichia coli* O157:H7 live as commensal species in the digestive track system of ruminants without causing any visible symptoms to the host (Wells et al., 1991; Chapman et al., 1993). The bacterium is excreted via feces and can survive in manure for over a long period of time. The most important STEC serotypes causing disease in humans are O157:H7, O26:H11, O103:H2, O145:H28, and O111:H8 (Pearce et al., 2004, 2006). In North America, Japan and Europe, O157:H7 is the type causing most disease incidences in humans and this is the reason why most research is focussed on this serotype. However, other serotypes like O26, O103, and O145 were regularly found in cattle from Scotland and USA (Wells et al., 1991; Wieler et al., 1996; Pearce et al., 2004, 2006; Shaw et al., 2004). Shiga toxin-producing O104:H4 or other entero-aggregative *Escherichia coli *strains were not found in cattle manure from Northern Germany during the Hamburg outbreak in 2011, indicating that cattle is not a major reservoir for the entero-aggregative* Escherichia coli* pathotype, to which the *Escherichia coli* O104:H4 outbreak strain belong to (Wieler et al., 2011).

The occurrence of *Escherichia coli* O157:H7 is more common in the intestinal track systems of cattle than in that of other ruminants and farm animals (Karmali et al., 2010; Ferens and Hovde, 2011 and references therein). The distribution of the pathogen in cattle feces is heterogeneous and occasionally numbers can be very high in excrements of particular individuals within the herd, so called super shedders (Ferens and Hovde, 2011). Epidemiological research on *Escherichia coli* O157:H7 infections in livestock in slaughterhouses in the Netherlands revealed prevalence of 10.6% (*n* = 540) in adult cattle, 0.5% (*n* = 397) in veal calves, 3.8% (*n* = 52) in sheep, 4.1% (*n* = 49) in lambs, 1.4% (*n* = 145) in pigs, 0% (*n* = 501) in chicken and 1.3% (*n* = 459) in turkey (Heuvelink et al., 1998a, 1999). Highest incidences in serotype O157:H7 contamination was thus found in adult cattle, but this serotype was also present in carcasses of other animals, with the exception of chicken. Examination of 10 dairy farms in the Netherlands, of which five were demonstrated to be serotype O157:H7 positive in their cattle, resulted in seven farms where at least one serotype O157:H7-positive individual was identified to be present (Heuvelink et al., 1998a). Within-herd prevalence on these seven farms varied between 0.8 and 22.4%. Surveillance of *Escherichia coli* O157:H7 in pooled fecal samples over 1051 dairy herds in the years 1997–2005 revealed on average prevalence of 8.0% (variation between 6.4 and 9.6%; Berends et al., 2008). In the same study surveillance of two distinct types of veal herds (so called pink and white veal herds) revealed clear differences between both types, i.e., 107 positive of 269 examined pink herds (39.8%) and 10 positive of 661 examined white herds (1.5%; Berends et al., 2008). The reason for this difference in infection prevalence was not further investigated, but may be related to difference in age before slaughtering, which is 35 weeks for pink veal and 25 weeks for white veal. Interestingly, screening of Dutch dairy farms on the basis of presence of Shiga toxin genes revealed much higher prevalence (80%) than via screening on serotype O157:H7 determinants (Franz et al., 2007). This would indicate that Shiga toxin genes, which are considered to be major virulence determinants in enterohemorrhagic *Escherichia coli* (EHEC), are not only present in serotype O157:H7, but can also be present in other serotypes as well. One important observation in the longitudinal study done in the Netherlands was the fact that the number of serotype O157:H7 in positive cattle herds shifted during the season showing maximum peaks in the second half of the summer period, whereas this serotype was undetectable in samples collected in wintertime (Heuvelink et al., 1998b; Bouwknegt et al., 2004; Schouten et al., 2004, 2005; Valkenburgh et al., 2007). Such a fluctuation during the season was also observed in other countries (Hancock et al., 1994; Chapman et al., 1997; Conedera et al., 2001; Barkocy-Gallagher et al., 2003; Milnes et al., 2009). This would indicate that fluctuations in serotype O157:H7, and possibly also in other serotypes, exist in cattle manure, which has a consequence in the control of serotype O157:H7 contamination of vegetable plants grown in manure-amended soils. Depending on the period in the season, contamination with pathogenic *Escherichia coli* serotypes expectedly will vary.

It was hypothesized that *Escherichia coli* O157:H7 is better adapted to circumstances prevailing in habitats outside of the digestive track of cattle than other non-Shiga-toxin producing* Escherichia coli* serotypes (Durso et al., 2004). This study revealed that a higher incidence of competitive strains were found among *Escherichia coli* O157:H7 than over non-Shiga toxin producing serotypes, but the non-Shiga toxin types were able to utilize a broader spectrum of tested substrates than *Escherichia coli* O157:H7. There were no other factors that could explain better fitness of *Escherichia coli* O157:H7 over non-Shiga toxin producing serotypes in alternative habitats, in spite of their relatively larger genome sizes that might be indicative for better adaptation to multiple habitats. Other, unknown factors therefore must be held responsible for survival of *Escherichia coli* O157:H7 outside the intestinal track systems of cattle.

The most likely route of contamination from cattle to plants is via manure through soil to plant roots, but other routes may exist like irrigation water (Erickson et al., 2010), flies (Talley et al., 2009) and equipment used by field workers (Johnston et al., 2009). Different transmission routes of *Escherichia coli* O157:H7 to arable plants may exist and remarkable is the rather long-term persistence of these strains in plant and soil ecosystems. It seems that at least some of the STEC strains and strains of other human pathogens like *Salmonella, Campylobacter*, and *Listeria* are better adapted to the circumstances prevailing in plants and soils than ever thought before.

### TRANSMISSION ROUTES OF HUMAN PATHOGENS IN PLANT PRODUCTION FIELDS

One of the first reported disease outbreaks caused by *Escherichia coli* O157:H7 and associated with consumption of vegetables was from potatoes in the UK (Morgan et al., 1988; **Table 1**). In this particular case, it was potato tubers that were suspected to be contaminated with *Escherichia coli* O157:H7 most likely originating from cattle manure and applied at potato production in the field. Because potatoes are not eaten raw, it was postulated that the transmission route of the pathogen was not via potato consumption itself, but rather from hand-to-mouth transfer at food preparation and handling of potatoes. From this case it became clear that the pathogen was able to persist over longer periods of time in soil near or at the surface of potato tubers. Potato lots containing the pathogen were not traceable anymore, so it is unknown at which densities and locations on, or may be inside tubers the human pathogen was present (Morgan et al., 1988). Concerning internalization, invasiveness of the pathogen would depend on specific enzymes like endoglucanases to create openings in plant cells, like plant pathogens do, or they would enter plants via natural openings (stomata and hydathodes) or woundings caused by feeding insects or nematodes or via co-infections with plant pathogens or soil-indigenous micro-organisms. The underground route of *Escherichia coli* O157:H7 and other human pathogenic enterics to plants will be discussed below as it is often hypothesized, but not always accepted as a realistic transmission route to plants.

#### Survival of human pathogens in manure-amended soils

The underground route of transmission of *Escherichia coli* O157:H7 to plants can be plausible if the survival time of the pathogen in manure-amended soil is long enough to allow successful colonization of plants. Under experimental circumstances, *Escherichia coli* O157:H7, introduced into autoclaved soils and mixed at different ratios with manure, was able to survive for more than 226 days at 15 and 21°C (Jiang et al., 2002). Lowering the temperature to 5°C led to a reduction in survival time to down to 35 days. Introduction into non-autoclaved manure-amended soil reduced survival to a maximal time of 193 days at 15 and 21°C, but there was no reduction in survival time at 5°C. This indicates that temperature is an important factor directly affecting survival time of the pathogen in soil, but also indirectly by influencing the activities of microbial populations resident in soil that antagonizes the invasive human pathogen. Higher average temperatures and higher oscillation amplitudes resulted in faster decline of both populations (Semenov et al., 2007). However, lower temperatures, between 10 and 15°C, commonly occur in temperate regions where incidences with *Escherichia coli* O157:H7 outbreaks are highest. Soil horizons where plant roots grow are often more constant in temperature than topsoil horizons and therefore, invasion of human pathogens to deeper soil layers would favor longer-term persistence in soil and thus increasing their chances on contact with plant roots. The effect of manure type and application to soil on percolation to deeper soil layers of the same *Escherichia coli* O157:H7 and *S. enterica* serovar Typhimurium strains were investigated in another study (Semenov et al., 2009). Both strains percolated to deeper layers (maximal studied depth was 40 cm) within a few hours after introduction, independent of manure type, and application of slurry resulted in percolation of both pathogens to greater depths than of solid manure, whereas the mode of application (spreading over soil surface versus injection into topsoil) had smaller effects on percolation depths of both pathogens. Percolation to deeper soil layers in fields may have two consequences for transmission to crop plants: one, that survival time may be increased because of the lower and more constant temperature present in deeper soil layers, and two, ground water may become contaminated which can be used for irrigation of crop plants (Brennan et al., 2010).

Available nutrients in manure and soil also were demonstrated to play important roles in survival of *Escherichia coli* O157:H7 and *S. enterica* serovar Typhimurium strains (Franz and Van Bruggen, 2008). Survival of *Escherichia coli* O157:H7 across 36 different manure-amended soils was investigated and it was shown that survival increased when slurry or chemical fertilizer (relatively rich in available C and/or N) were applied to soils in comparison with applications of farmyard manure or compost (relatively poor in available C and N). Therefore, it was concluded that circumstances characterized to be oligotrophic for bacteria decreased *Escherichia coli* O157:H7 survival in manure-amended soils. Manure type itself is also another factor influencing survival of human pathogens in manure-amended soils. Manure type depends on the diets that cows had received and the effect of roughage of the diet on the fate of introduced *Escherichia coli* O157:H7 and *S. enterica* serovar Typhimurium strains in manure was investigated (Franz et al., 2005). Population decline of introduced *Escherichia coli* O157:H7 was fastest in manure from cows fed with the roughest diet type (straw). The effect of roughage of the diet on *S. enterica* serovar Typhimurium survival in manure was less clear than for* Escherichia coli* O157:H7, although decline rates of both pathogens were fastest in manures of cows fed with the roughest diet. Fiber content and pH of manure were the best explanatory factors for decline rates of both pathogens.

The nutrient status in manure-amended soils is a factor that plays a direct role in survival of* Escherichia coli* O157:H7 and other human pathogens. However, indirectly, the nutrient level also may play a role in the diversity and evenness of microbial populations that are indigenous to manure-amended soil that might compete with invasive human pathogens (Semenov et al., 2008; Van Overbeek et al., 2010). Soil bacterial diversity as an experimental variable indeed was shown to be negatively correlated with longevity in survival of an introduced *Escherichia coli* O157:H7 strain, representing a species invasive to soil (van Elsas et al., 2012). These data all together illustrate the importance of high microbial diversity to reduce survival time of human pathogens as species invasive to soils.

Finally, survival time is dependent on strain differences, even within the same species. Remarkable differences in survival time between different *Escherichia coli* O157:H7 strains was observed among 18 different strains of which eight originated from animal, one from food, and nine from human sources (Franz et al., 2011). Survival time in manure-amended soils of the nine human strains were longer than of the other nine strains. Principle component analyses on metabolic profiles of all 18 strains revealed separate clustering of the human strains from the others and the ability of the nine human strains to oxidize propionic acid, α-ketobutyric acid and α-hydroxybutyric acid appeared to be discriminative for this particular environmental group. It was therefore concluded that phenotypic diversity found among different *Escherichia coli* O157:H7 strains may explain observed differences in survival time in manure-amended soils. Differences in survival time among different strains of *Escherichia coli* O157:H7 would indicate that some strains are better adapted to circumstances prevailing in natural habitats, like soils, than others. Presence or absence of important virulence factors in all 18 *Escherichia coli* O157:H7 strains appeared not to play any role in survival time in manure-amended soils. This is in line with observations made in a study done on different *stx*1, *stx*2, both *stx* and *eae *(intimin) gene deletion mutants made from *Escherichia coli* O157:H7 EDL933 strain in soils differing in texture (loamy sand, sandy loam, silty clay; Ma et al., 2011). There are thus no indications that virulence to humans has an effect on *Escherichia coli* O157:H7 survival time in (manure-amended) soils. However, mutation in an important gene involved in stress regulation, *rpo*S, had an effect on survival of *Escherichia coli* O157:H7, leading to reduced persistence in manure-amended soil (van Hoek et al., 2013).

#### Plant colonization by human pathogens

Presence of human pathogens in contaminated soil at high cell densities may lead to plant internalization. This was demonstrated in lettuce plants grown in soil amended with cow manure containing high doses (10^6^ and 10^8^ CFU/g soil) of a GFP-marked *Escherichia coli* O157:H7 strain (Solomon et al., 2002). The introduced strain was retrieved from lettuce seedlings even after 10 min treatments with HgCl_2_, indicating that internalization of plant tissue by the pathogen had occurred. Confocal microscopy on lettuce leafs colonized by the GFP-marked *Escherichia coli* O157:H7 strain revealed the presence of fluorescent aggregates in intercellular spaces. There was no evidence for intracellular colonization by *Escherichia coli* O157:H7, but the presence of the pathogens inside plant tissue of the edible parts of the plant already indicate that there is a risk upon consumption of fresh produce, because cells cannot easily be removed or inactivated by washing and disinfection procedures. Adherence and internalization of plant tissue are consequences when human pathogens come into contact with plants.

Longer-term survival of human pathogens in manure-amended soils ultimately will lead to lower inoculum levels in soils and thus to lower risks of crop plants to become contaminated. The question that arises is whether human pathogens are still capable to contaminate plants after longer periods of residence in soils. A mixture (1:1) of *S. enterica* (serovars Baildon and Enteritidis) strains was detectable to up to 6 weeks after introduction into soil (Barak and Liang, 2008). Sowing tomato seeds at weekly intervals after inoculation of this mixture revealed the presence of the pathogen mixture in the rhizoplane and phylosphere of plants for up to 7 weeks after inoculation. Inoculation of soils with *Escherichia coli* O157:H7 via three different composted manure types and irrigation water revealed the presence of the pathogen strain in lettuce and parsley plants for up to, respectively, 77 and 177 days after transplanting (Islam et al., 2004a). Using the same carriers, *S. enterica* serovar Typhimurium was detectable in radish and carrots after, respectively 84 and 203 days after sowing of seeds into soils (Islam et al., 2004b). No clear effects of the carrier types for the *Escherichia coli* O157:H7 and *S. enterica* serovar Typhimurium inoculants were found in these studies, however, longevity of survival differed among different tested plant species, indicating that there was an effect of plant type on survival of both pathogen strains. *Escherichia coli* O157:H7 and *S. enterica* serovar Typhimurium strains inoculated at levels of 10^7^ CFU per g manure-amended soil were retrieved from the rhizosphere (Ongeng et al., 2011a) and internal compartments (Ongeng et al., 2011b) of transplanted cabbage (*Brassica oleracea*) plants grown under tropical field conditions. These two studies indicated that the presence of plant roots may extend survival time of human pathogens in soils and that internalization of crop plants may occur following successful rhizosphere colonization.

Long-term persistence in soil may lead to nutrient starvation of human pathogens in soil. These pathogens may gear to forms that are more resistant against stresses, although they also may become metabolically arrested, entering the so called viable-but non-culturable (VBNC) state as was demonstrated for another gammaproteobacterial species, *P. fluorescens* (Van Overbeek et al., 1995). Cells of the *Escherichia coli* O104:H4 Hamburg-outbreak strain (**Table 1**) imposed to copper and low temperature as stress factors became VBNC as demonstrated by viability staining of non-culturable cells (Aurass et al., 2011). However, non-culturability most likely did not affect virulence, as genes responsible for virulence appeared to remain intact. This to the contrary to a typical soil-borne plant pathogenic bacterium, *Ralstonia solanacearum* biovar 2, that also became VBNC upon treatment at low temperature, but these cells apparently lost their virulence upon injection into host (tomato) plants, where these cells still were capable to multiply, demonstrating that they were resuscitated from the VBNC state (van Overbeek et al., 2004). Exposure of *S. enterica *serovar Typhimurium LT2 to cold stress (5 h at 5°C) resulted in higher resistance to acid (pH 4.0) stress (Shah et al., 2013). Persistence of *S. enterica *serovar Typhimurium DT104 in soil and lettuce plants resulted in better survival in simulated gastric fluid than cells freshly grown in culture, however, these cells were less capable to attach and invade epithelial cells (Oliveira et al., 2011). Observed effects slightly differed between the two studied strains (one originating from pig carcass and the other from lettuce plants), but illustrates that physiological aspects in cells of human pathogens can play a role in adaptation to circumstances prevailing in the phytosphere, possibly leading to increased transmission to humans upon consumption.

Presence of plant roots in soil play an important role in the entrance of human pathogens into plants. During root colonization, human pathogens must successfully compete with rhizosphere-indigenous micro-organisms for available nutrients. Iron is an essential element for bacteria and a limiting factor for growth of bacteria in the rhizosphere. Mutations in a precursor in siderophore production, or in siderophore production itself, in *S. enterica* serovar Typhimurium resulted in a significant lower colonization of alfalfa roots (Hao et al., 2012). Siderophore production in *S. enterica* is essential for root colonization, as it is typical for rhizosphere bacteria like *P. fluorescens* and *R. solanacearum*. Siderophore production is a colonization fitness factor for *S. enterica* (Hao et al., 2012) and this would indicate that the pathogen is well-adapted to circumstances prevailing in the rhizosphere.

However, human pathogens contaminating vegetable plants do not necessarily originate from soil, but may originate from seeds, as seeds were supposed to be the main contamination route for the *Escherichia coli* O104:H4 outbreak strain to fenugreek sprouts at the Hamburg outbreak incidence in 2011*. S. enterica* and *Escherichia coli* O157:H7 were both shown to persist on lettuce seeds for 2 years and still capable to colonize young lettuce plants (van der Linden et al., 2013). Of both human pathogens,* S. enterica* was shown to be the best survivor on lettuce seeds. These examples show that contamination of arable plants can occur via different routes and that long-term persistence in soil or on dry seeds both can be important factors in successful colonization of plants.

#### Proposed mechanisms of plant internalization by human pathogens

Internalization of plants by human pathogens may result from passive or active processes. Cells of *Escherichia coli* O157:H7 “Sakai” strain, introduced to spinach and lettuce plants under experimental conditions using a high inoculum dose of 2 × 10^7^ CFU per ml, were shown to be present inside root tissue of both plant species (Wright et al., 2013), indicating that this human pathogen potentially can enter these plants by itself. It may be assumed that human pathogens do not possess the same specific traits, like cell wall degrading (pectinolytic) enzymes present in plant pathogens that are required for efficient invasion of plants. The presence of plant pathogens at the same locations on the plant surface where human pathogens reside would facilitate entrance of human pathogens into plants as was proposed in Teplitski et al. (2009, 2011). Under practical circumstances in agronomic systems, other soil species including plant pathogens indeed appeared to play important roles in spread, colonization, and internalization of plants by human pathogens. The bacterivorous nematode *Caenorhabditis elegans* was demonstrated to transport *S. enterica* serovar Newport, initially introduced into manure, through manure-amended soil to lettuce, strawberry and carrot plants (Kenney et al., 2006). *Xanthomonas campestris* pathovar vesicatoria facilitated a mixture of *S. enterica* strains to colonize the tomato phytosphere (Barak and Liang, 2008). Soft rot caused by *Pectobacterium chrysanthemi* in postharvest lettuce resulted in higher density levels of *Escherichia coli *O157:H7 in lettuce leafs (Brandl, 2008) and coinoculation of *S. enterica* serovar Typhimurium with the plant pathogen *Dickeya dadantii* under experimental conditions in lettuce and cilantro leaves resulted in higher densities of *S. enterica* serovar Typhimurium in leafs of both plant species than by inoculation with the human pathogenic strain alone (Goudeau et al., 2013). However, the presence of the root knot nematode *Meloidogyne hapla* in soil with bioluminescent-labeled *Escherichia coli* O157:H7 cells did not result in colonization of the areal parts of young spinach plants (Hora et al., 2005). Mechanic wounding of roots and coinoculation of leafs with the *Escherichia coli* O157:H7 strain and *Pseudomonas syringae* also did not result in colonization of the aerial parts of spinach plants, indicating that not all plant pathogens are facilitating entrance of human pathogens into, and/or spread through plants. Other species living in soil, like protozoa and fungi, may play an important role in the spread of *S. enterica* through soil and colonization of plants (Brandl et al., 2013) and it was demonstrated that *S. enterica* also can live inside protozoa cells (Jacobsen and Bech, 2012, and references therein). All together, these studies indicate that presence of plant pathogens and other species living in soil can play different roles in colonization and internalization of plants by human pathogens. Interesting within this aspect is that *Enterobacter* sp. 638, an endophyte in poplar tree, is capable to degrade pectate, facilitating this strain to colonize spaces between plant cells (Taghavi et al., 2010). Considering its close taxonomical relatedness with *Escherichia coli* and *S. enterica*, it emphasizes the fact that specific traits required for plant colonization must have been acquired by particular *Enterobacteriaceae* during evolution near or inside plants.

*Enterobacteriaceae* are common inhabitants of the phytosphere. To this taxonomical family belong important plant pathogens like *Erwinia, Pantoea,* and *Pectobacterium* species (Holden et al., 2009; Teplitski et al., 2009, 2011). Of more importance are genera whose species are beneficial to plants and pathogenic to animals, such as the ones belonging to *Serratia* and *Klebsiella* (Berg, 2000; Tan et al., 2001; Dong et al., 2003; Tyler and Triplett, 2008; Holden et al., 2009). Plant and animal-associated enteric species share genes that encode for important virulence traits such as attachment, plant colonization and internalization, biofilm formation and cell invasion (Tyler and Triplett, 2008; Holden et al., 2009; Teplitski et al., 2011). The taxonomic relatedness between human pathogens and plant-associated species may explain why at least some of the human pathogenic strains can colonize plants so well. Ancestors of both groups diversified along evolution, but parts of their genomes, so called cores, remained the same and possibly some of the orthologous genes shared by both groups may be involved in plant interactions (Holden et al., 2009).

Attachment to plant surfaces is an important feature related with settlement of human pathogens to, or inside plants. Constituents of the extracellular matrix of human pathogens like curli fibers, cellulose and lipopolysaccharide capsule (O antigen) were important for attachment to, and colonization of alfalfa sprouts by *S. enterica* serovar Newport (Barak et al., 2007) and spinach leafs by *Escherichia coli* O157:H7 (Macarisin et al., 2012). Possible mutations in regulatory genes responsible for curli and/or cellulose fiber production and leading to the non-rdar morphotype (defective in the formation of red dry and rough colonies on Congo red agar plates; Römling, 2005) resulted in better fitness of *S. enterica* serovar Typhimurium inside tomato fruits (Gu et al., 2011; Zaragoza et al., 2012). The same phenotype in *S. enterica* was less competitive in growth medium in comparison with its near isogenic wildtype strain (Zaragoza et al., 2012). Particular subsets of strains of human pathogens appear to possess features enabling them to attach to plant surfaces.

Entrance of human pathogens via aerial plant parts like woundings or natural openings such as hydathodes or stomata is realistic. A GFP-labeled *S. enterica* serovar Typhimurium strain was attracted to the stomatal openings of iceberg lettuce leafs under the influence of light, where it entered the stomatal cavity (Kroupitski et al., 2009). This strain was able to circumvent the stoma-based innate immune system. Internalization of *Escherichia coli* O157:H7 via stomata was also demonstrated in spinach leafs (Saldaña et al., 2011). Reduced cell numbers of a type 3 secretion defective mutant of this strain was found in the stomatal opening, in comparison with the wild type strain, indicating that type 3 secretion must play an important role in internal colonization of plants. Transfer of the locus of enterocyte effacement (LEE) pathogenicity island, containing type 3 secretion effector genes, of the *Escherichia coli* O157:H7 strain into a non-pathogenic *Escherichia coli* K12 strain resulted in improved colonization of derived strain. Both *S. enterica* and *Escherichia coli* O157:H7 possess mechanisms to actively enter the apoplast, thereby circumventing plant host immunity responses. Colonization of the interior parts of Roman lettuce by *S. enterica* serovar Dublin strain evoked an upshift in the expression of stress-related plant genes (Klerks et al., 2007b). Of the five different *S. enterica* serovars tested (Dublin, Typhimurium, Enteritidis, Newport and Montevideo), the strain of serovar Dublin was demonstrated to be the best colonizer of Roman lettuce, indicating the existence of differences in plant colonizing traits among different *S. enterica* serovars (Klerks et al., 2007a). *S. enterica* serovar Dublin strain was attracted to sugar-like carbon sources present in root exudates of lettuce and these compounds were responsible for induction of different genes amongst which genes that are regulators of the type 3 secretion system (Klerks et al., 2007a). *S. enterica* serovars actively colonize plants and expression of genes under type 3 secretion control also appeared to play an important role in the suppression of the plant immune system (Schikora et al., 2012).

Once inside plants, human pathogens may colonize plants locally, but also may systemically spread through plants by making use of the vascular tissues present in plants and needed for the transport of water and inorganic substances to leafs (xylem) or photosynthates to roots (phloem; McCully, 2001; Warriner et al., 2003). Five different *S. enterica* serovar strains, applied to plants via inoculation of the flowers, were later found to be present in tomato fruit by Guo et al. (2001). Systemic transport to aerial parts of tomato plants occurred upon growth in hydroponic solution containing the five different *S. enterica* serovar strains at levels of between 10^4^ and 10^5^ cell per ml (Guo et al., 2002). From the last study there are clear indications that transport inside tomato plants to developing leafs and branches must have occurred. Presence of *S. enterica* serovar Newport and *Escherichia coli* O157:H7 strains in chaff and seed of Arabidopsis plants was demonstrated when plants were grown under gnotobiotic circumstances and in autoclaved and non-autoclaved soils (Cooley et al., 2003). All together, the last two studies are indicative for systemic transport of human pathogens through vascular tissue, but no conclusive data could be provided yet. Vascular transport may occur in low quantities, but numbers may be too low to be detectable with common technologies (Warriner and Namvar, 2010).

In conclusion, it is likely to assume that features in particular subsets of *Escherichia coli* and *S. enterica* groups exist, enabling them to persist near or inside plants over extended periods in time.****Genes in human pathogens involved in persistence in plants may be orthologs, but it cannot be ruled out that at least some of these genes were also recently gained via horizontal gene transfer, as was demonstrated to be the case for the *Escherichia coli* O104:H4 outbreak strain in Hamburg and surrounding area in 2011. In modern agriculture, practices are applied to optimize crop yields by irrigation, fertilization, pest, and disease control and maintenance of soil quality. This requires input from different sources, and bacterial communities from plants themselves, soils, intestinal track systems of farm animals (manure), water reservoirs (for sprinkling and irrigation of plants) and agricultural runoff water (Jacobsen and Bech, 2012) all come together at plant production (**Figure 1**). Human pathogens or their phages (examples are provided later) were found to be present in all four ecosystems, potentially bringing these into contact with indigenous bacteria near or inside plants. The consequences of these contacts can be exchanges in genetic material and the likelihood on occurrence of genetic exchanges between human pathogens and plant-associated bacteria and potential risks resulting from these will be discussed in the following section.

## HORIZONTAL GENE TRANSFER BETWEEN HUMAN PATHOGENS AND THEIR (PRO) PHAGES IN THE PHYTOSPHERE

### HISTORICAL OCCURRENCES OF GENE EXCHANGES IN TWO MAJOR HUMAN PATHOGENS, *Escherichia* COLI SEROTYPES O104:H4 AND O157:H7

Gene transfer can occur when bacterial cells physically meet within the same habitat (Toth et al., 2006). Proposed vehicles for transmission of genomic islands are plasmids, (pro) phages and conjugative transposons (Juhas et al., 2009). Acquisition of new phenotypic traits will occur when human pathogens reside outside the human host. The plant-soil ecosystem is such an environment where these species can occur and thus it is likely to assume that auxiliary traits will be acquired from the microbial communities indigenous to this ecosystem.

*Escherichia coli* strain O104:H4, the outbreak strain in Germany and France in 2011, substantially differed from *Escherichia coli* O157:H7 outbreak strains (Bielaszewska et al., 2011; Eppinger et al., 2011; Mellmann et al., 2011). Some characteristics of the outbreak strain were: (i) that it belonged to the pathotype of enteroaggregative *Escherichia coli* and not to that of enterohemorrhagic *Escherichia coli*, (ii) that it did not produce intimin (encoded by the *eae* gene located on the LEE) but instead Iha adhesin and (iii) that it only produced Shiga toxin 2. The strong adherence of the outbreak strain to the intestinal epithelium in humans in combination with high tolerance to acid (to survive passage through the stomach) and Shiga toxin production were believed to be the main responsible factors for the occurrence of high incidence in HUS in patients, stressing the risks for humans by blends of virulence factors that can occur among human pathogens (Bielaszewska et al., 2011). These differences can be explained by differences in the nature of both serotypes, but also in genomic changes resulting from gene insertions and deletions. The LEE in serotype O157:H7 strains is a conserved pathogenicity island containing genes that are coding for chaperone and effector proteins, belonging to the type 3 secretion system, and responsible for the attaching and effacing lesions in the human large intestine. Intimine is a protein that is involved in cellular attachment of O157:H7serotype strains and the gene coding for this protein (*eae*) was not present in serotype O104:H4 as well as in other entero-aggregative *Escherichia coli* strains.

*Escherichia coli* O157:H7 strains commonly possess a virulence plasmid (pO157) and a conjugal plasmid (pEC4115; Eppinger et al., 2011), whereas in both serotypes, O104:H4 and O157:H7, *stx* genes are located on lambdoid prophages integrated in the *wrb*A gene (Mellmann et al., 2011). The genome of the serotype O157 Sakai strain possessed a total of 18 prophages, of which two contained the Shiga toxin genes *stx*1 and *stx*2 (Asadulghani et al., 2009). Despite the fact that these prophages contained multiple mutations, they were still functional, indicating that these genetic elements were able to propagate and to recombine with other genetic elements. The roles of phages that did not carry *stx* genes were inferred from complete genome sequence and comparative genome analysis of *Escherichia coli* strains (Ogura et al., 2008; Iguchi et al., 2009). It revealed that virulence genes on exchangeable effector loci were present that code for non-LEE and LEE effectors belonging to the type 3 secretion system. Some of these genes harbor homology to effector proteins of plant pathogens, strengthening the idea of a common evolutionary plan for the type 3 secretion system.

An evolutionary model of the serotype O104:H4 outbreak strain was constructed, based on available whole genome sequence data from a proposed ancestor strain, another *Escherichia coli* O104:H4 strain from an outbreak in 2001 (Mellmann et al., 2011). Interesting to note is that the German *Escherichia coli* O104:H4 outbreak strain of 2011 must have acquired the plasmid containing the aggregative adherence fimbriae type I (AAF/I) locus, but lost a plasmid containing the AAF/III locus and gained a plasmid with the gene encoding CTX-M-15 ESBL.

Virulence traits were gained, likely via horizontal gene transfer, and it is still under debate whether plasmid or phage exchanges between human pathogens and commensal species will occur under changing environmental circumstances. In a model for genome evolution proposed by Dini-Andreote et al. (2012), it was postulated that bacterial genomes will tend to expand under situations of shifting environmental conditions and that genomes shrink under stable environmental conditions. Translating this model for our study, it means that genomes of pathogens that occupy single habitats, like inside human bodies, will shrink whereas the ones that are exposed to multiple habitats will tend to increase in size, which will be the case for environmental strains. Proposed increase in genome size may occur by acquisition of new genes via gene transfer events. The “pan-genome” encompasses all genes that are present among strains originating from different environments, but still belonging the same taxonomic group (Dini-Andreote et al., 2012). It is characteristic for genomes of enterohemorrhagic species that stretches of housekeeping genes, forming the core genome, are punctuated by gene islands, forming the flexible genome (Mellmann et al., 2009). Genes belonging to the flexible genome most easily will be exchanged between bacteria of the same species, but also between bacteria of sometimes entirely different taxonomic groups. The flexible gene pool will consist of gene clusters, so called genomic islands that are responsible for auxiliary traits. The formation of genomic islands in bacterial genomes will facilitate the transfer of new phenotypic traits to recipients, resulting in “quantum leaps” in the adaptation of receiving strains to new environments (Juhas et al., 2009).

### HORIZONTAL GENE TRANSFER BY BACTERIOPHAGES

Bacteriophages play a role in the virulence of EHEC strains by conferring genes involved in toxin production and type 3 secretion. Bacteriophages are recognized to be the main contributors to the transmission of virulence determinants, via transduction and lysogenic conversion, between bacterial strains. This process of lateral gene transfer is a significant factor in the evolution of bacteria. DNA of bacteriophage origin often comprises 10–20% of a bacterial genome, and approximately two-third of gammaproteobacterial and low-GC Gram-positive species harbor intact bacteriophages or their remnants in their genomes (Davidson et al., 1990; Saye et al., 1990; Kidambi et al., 1994; Boyd et al., 2000; Perna et al., 2001; Wagner and Waldor, 2002; Canchaya et al., 2003a,b; Casjens, 2003; Toth et al., 2003). A recent metagenomics survey on the virome of the bovine rumen showed that 64% of known virus genomes are from bacteriophages (Berg Miller et al., 2012). In particular, prophages were shown to originate from bacterial hosts within the taxonomical groups of *Firmicutes* (68 ± 1%), *Proteobacteria* (18 ± 1%) and *Bacteriodetes* (8 ± 1%). Comparison of the bovine rumen virome with a selected set of mobile elements from microbial genomes of the bovine rumen revealed a similarity of 80 ± 19%. The vast majority (79 ± 19%) was similar to prophages, demonstrating the importance of this type of mobile element within this ecosystem.

Shiga toxins, encoded by *stx*1 and *stx* 2 genes, are located on temperate bacteriophages and low or avirulent forms of *Escherichia coli* can be converted to pathogenic strains upon infection with these phages. Bacterial conversion by phages from non-pathogenic to pathogenic forms occur more often, for example in *Corynebacterium diphtheria* and in different *Salmonella* species (Saunders et al., 2001). Bacterial phages are responsible for horizontal transfer of virulence determinants between different bacterial species. Bacteriophage Φ24_B_, isolated from an enterohemorrhagic *Escherichia coli* O157 strain and carrying the *stx*2 gene, was able to infect commensal and pathogenic *Escherichia coli* and *Shigella* strains from different sources (James et al., 2001). This indicates that the host-range of this bacteriophage is broader, conferring new virulence traits toward different species among the group of *Enterobacteriaceae*. Expression of Shiga toxin genes are controlled by lambdoid prophage cI repressor (Mauro and Koudelka, 2011). Shiga toxin gene expression is stimulated upon cleavage of cI by RecA, and on its turn RecA expression can be stimulated by the presence of reactive oxygen species released from eukaryotic predators like *Tetrahymena thermophila* (Lainhart et al., 2009; Mauro and Koudelka, 2011). Shiga toxin production is a virulence determinant whose contribution to bacterial fitness is obscure. As discussed before, knock out mutants in Shiga toxin production did not survive better or worse in soils and plants. A question that still remains open is the role of Shiga toxin production in bacterial survival in the environment. Shiga toxin production may be a defense mechanism against eukaryotic unicellular predators, suggesting that humans may not be the prime targets for Shiga-toxin producing *Escherichia coli* strains.

A total of 15 phages carrying Shiga toxin *stx*2 genes, purified from environmental water samples, were shown to belong to three different virus families: *Podoviridae* (9), *Myoviridae* (6) and *Siphidoviridae*, the family to which phage lambda belong (2; Rooks et al., 2012). Environmental water thus can be a source of *stx*-carying phages and transduction with this type of phages may increase virulence levels in *Escherichia coli* strains. A *Siphidoviridae* lytic phage targeting the plant pathogenic bacterium *Pectobacterium carotovorum* subsp. *carotovorum*, My1, was recently sequenced (Lee et al., 2012). Although no *stx* genes were found in the genome of this phage, it illustrates the possibility that lytic phages released from plant-pathogenic bacteria can infect plant-invasive *Escherichia coli* strains. Broad-host range lytic phages have been characterized, like phage ΦOT8 from treated sewage effluent, that was able to infect and tranduce resistance and prodigiosin marker genes from *Serratia* sp. ATCC 39006 to *Pantoea agglomerans* (Evans et al., 2010). Phages isolated from soil and trees from Ontario, Canada (Gill et al., 2003) and from apple and pear orchard soils in Switzerland (Born et al., 2011) were able to infect the plant pathogenic bacterium *Erwinia amylovora*. All bacteriophages obtained (42 from the study of Gill et al., 2003 and 24 of the one of Born et al., 2011) belonged to *Podoviridae* or *Myoviridae*. Commonalities in genome sequence were found between two *Myoviridae* phages from both studies, ΦEa21-4 and M7, and both were considered to be broad-host range phages infecting three different *Erwinia* species and *Pantoea agglomerans* (Born et al., 2011). The same phage type (ΦEa21-4) showed a remarkable similarity at a protein level of 56% with *Salmonella* phage Felix O1 (Lehman et al., 2009). Major difference between phages ΦEa21-4 and Felix O1 was the presence of the *nad*V homolog encoding a nicotinamide-scavenging enzyme that may supplement nicotinic acid in *E. amylovora* (Lehman et al., 2009). Typical ΦEa21-4-like phages may be cosmopolites able to infect multiple species within the class of *Enterobacteriaceae*. In potential, this type of phages could also be able to horizontally transfer genes from and to human pathogens upon invasion into the phytosphere.

### HORIZONTAL GENE TRANSFER BY CONJUGAL PLASMIDS

Virulence traits are present on different plasmids in *Escherichia coli* (Johnson and Nolan, 2009). Commonalities exist in pathogenicity determinants among plant and animal *Enterobacteriaceae* (Toth et al., 2006). Therefore, conjugal plasmids can play pivoting roles in horizontal transmission of virulence factors located on pathogenicity islands in plant-soil ecosystems.

The Hamburg serotype O104:H4 outbreak strain contained TEM-1 and CTX-M-15 beta lactamase genes on an incompatibility group I (IncI) type plasmid (Mellmann et al., 2011). Extended spectrum beta lactamase phenotypes have been found in many enteric species over the world, and CTX-M15 was found in *Escherichia coli, Klebsiella pneumonia, Enterobacter aerogenes* and *S. marcescens* (Bonnet, 2004 and references therein). Further, CTX-M class of beta lactamase genes were found in *Erwinia persicina* (Vimont et al., 2002) and in *P. aeruginosa* and *S. maltophilia* (Naiemi et al., 2005). IncI-type plasmids are narrow host-range plasmids and were not transferrable between donor and recipient *Escherichia coli* strains in nutrient-amended soil (Pukall et al., 1996). In that study, broad-host range plasmids belonging to incompatibility groups IncN, IncP1, IncW3, and IncQ were transferred to the *Escherichia coli* recipient strain and it was hypothesized that size and flexibility of the pili covering the cellular surface of donor cells would play an important role in the frequency of plasmid transfer in soil (Pukall et al., 1996; Ghigo, 2001). IncP-type of plasmids are self-transmissible plasmids, whereas IncQ types are not, however, both were considered to be the major vectors in horizontal transfer of antibiotic resistances in natural environments (Heuer et al., 2012; Popowska and Krawczyk-Balska, 2013).

Virulence factors in human pathogens are often located on plasmids. Tellurite (TeO_3_) resistance is a trait that is commonly found among different human pathogens including *Escherichia coli* O157:H7 and O46:H^-^, and were located on a pathogenicity island (Taylor, 1999). Tellurite resistance genes in enteric bacteria, located on IncHI2 and IncHII plasmids, showed remarkable homology with tellurite resistance genes located on plasmid R478 from *S. marcescens* (Taylor et al., 2002). Tellurite resistance determinants were also found on a IncP2 plasmid from *P. aeruginosa*, but these genes were not related with the ones found in *Escherichia coli* O157:H7 (Hou and Taylor, 1994). Iron acquisition genes of *Yersinia* pathogens (*Y. pestis, Y. pseudotuberculosis, Y. enetrocolitica*) is located on the *Yersinia* high pathogenicity island (HPI) and this HPI was found among different species of *Enterobacteriaceae*, including *Escherichia coli*, *Citrobacter diversus*, different *Klebsiella* species, non-I serotypes of *S. enterica*, but not in *S. enterica* serovars Enteritidis and Typhimurium and in EHEC (Bach et al., 2000; Carniel, 2001). Mobility of the HPI among enteric species must occur, although in *Y. pseudotuberculosis* excision was shown to be a rare event (frequency of about 10^-9^; Carniel, 2001). An HPI found in *Escherichia coli* showed strong (99%) identity with HPI present in *Y. pestis*, indicating that gene transfer between *Y. pestis* and *Escherichia coli* must have occurred in the past (Schubert et al., 2004). Mobilization of HPI in the bacterial chromosome is a phage-mediated process and P4-phage-related integrase and excision genes were found to be present in HPI (Benedek and Schubert, 2007). However, promiscuous mobile plasmids play important roles as shuttle vectors in the horizontal transfer of HPI (Antonenka et al., 2005; Schubert et al., 2009) and other pathogenicity islands in *Escherichia coli* (Schneider et al., 2011) and in environmental and other pathogenic micro-organisms (Dobrindt et al., 2004). Upon introduction of the self-transmissible plasmid RP4 into *Y. pseudotuberculosis*, a cointegrate between this plasmid and HPI was formed and transferred to a recipient strain (Antonenka et al., 2005). Trapping of HPI into the cointegrate was based on precise excision of the element from the chromosome and the frequency in occurrence of this event was estimated at 1 on 10^6^ cells. Precise excision did not always occur, and occasionally regions flanking the HPI were also integrated and transferred to recipients (Schubert et al., 2009). *Salmonella* genomic island 1 (SGI1), conferring multiple antibiotic resistances to the bacterial host, was shown to be excised from the chromosome by a lambdoid integrase (Doublet et al., 2005). However, the circular form of SGI1 was not transferred to recipient strains by itself under experimental circumstances. A helper IncC plasmid (R55) was needed for the transfer of SGI1 from different *Salmonella* donor strains to *Escherichia coli* K12 as recipient (Doublet et al., 2005). A screening over 902 *S. enterica* serovar types from poultry revealed a higher incidence of a ColV type plasmid among a clonal type of serovar Kentucky than over other serovar types (Johnson et al., 2010). The ColV plasmid conferred virulence and fitness traits toward its host and strains carrying the plasmid better colonized the chicken cecum and successfully competed with the indigenous microbial community in there. This illustrates the ecological advantage that may be gained by human pathogens via transfer of genes located on mobile elements like plasmids. The genes present on plasmids in human pathogens may come from different sources as demonstrated by the full sequence of the large virulence plasmid, pO157, from *Escherichia coli* O157:H7 (Burland et al., 1998). A strong resemblance between genes located on this plasmid was found with ToxA and ToxB toxin protein genes from *Clostridium difficile*. Genes of archeal origin (presumably from *Methanobacterium thermoautotrophicum* and *Halobacterium* species) were proposed to be present in the genome of *Escherichia coli* O157:H7 strain EDL933 (Faguy, 2003). The last would indicate that cross-domain gene transfer events would have occurred in the past between *Escherichia coli* and *Archeae* species.

Besides transfer of genes belonging to the flexible genome (mostly located on genomic or pathogenicity islands), also genes of the core genome can be transferred like household and metabolic genes. Among these genes can be ones that improve fitness of the recipient strain under environmental circumstances like present in the rhizosphere. Transfer of metabolic genes involved in oxidation or fermentation of small carbohydrates like sugars, acids, and amino acids may improve fitness of receiving strains in the rhizosphere where these compounds are exuded by plant roots. Genes responsible for L-sorbose transport and metabolism in *Escherichia coli* was also found in *K. pneumonia* (Wehmeier et al., 1992) and genes involved in glucose fermentation were transferred via genetic elements among different *Salmonella* species (Wohlhieter et al., 1975; Bartlett and Trust, 1980). Only a few strains of *Salmonella* and *Escherichia coli* can ferment glucose and acquisition these genes can be important for adaptation to new environments. Transfer of sucrose fermenting genes was found to be facilitated by conjugative transposons (Pembroke et al., 2002).

Under experimental circumstances, transfer of an environmental (wastewater treatment plant) self-transmissible and broad-host-range (IncP1β) plasmid from *P. putida* to different *S. enterica *serovar and *Escherichia coli* O157:H7 strains was demonstrated (Van Meervenne et al., 2012). Upon bacterial mating between donor and recipient strains, transconjugants were found at most, but not all occasions, indicating that human pathogens are not equally susceptible for horizontal gene transfer events under applied conditions. Bacterial gene transfer between introduced plasmid donor and/or recipient strains was demonstrated at different places in the phytosphere, such as in the rhizosphere (Lilley and Bailey, 1997), the phylloplane (Normander et al., 1998) and inside plants between endophytes (Taghavi et al., 2005). Transfer of two plasmids from *P. putida* recipients to indigenous bacteria associated with ready-to-eat alfalfa sprouts was demonstrated (Mølbak et al., 2003). In this study, transconjugants were found among different *Pseudomonas* and *Erwinia* species. Mobilizable plasmids from indigenous bacteria of piggery manure were obtained by introduction of *Escherichia coli* and *P. putida* strains, acting as recipients for exogenous plasmid isolation, in samples from different ecosystems (Smalla et al., 2000). Identities of plasmids selected from transconjugants revealed that they were all IncQ like. IncQ and IncP, IncN, and IncW type plasmids carrying gentamycin (Heuer et al., 2002) or streptomycin (van Overbeek et al., 2002) resistance determinants were obtained by exogenous plasmid isolations from bulk and rhizosphere soils, manure, sludge, and seawater samples. These data demonstrate the omnipresence of mobilisable (like IncQ type) and sometimes self-transmissible (like IncP type) plasmids in a wide variety of ecosystems, including the ones that are relevant for agriculture. Remarkable was the high abundance of IncQ-type of plasmids obtained by exogenous plasmid isolation, because these plasmids need a helper plasmid from another incompatibility group for transfer. Transfer of IncQ plasmids would require three parents for mating and it was believed that these type of matings would occur less frequently in soils (Pukall et al., 1996).

Cryptic plasmids can play important roles in horizontal transfer of IncQ plasmids in natural ecosystems. A cryptic plasmid, pIPO2, was found in the rhizosphere of wheat and this plasmid was able to mobilize IncQ plasmids to recipient strains (Van Elsas et al., 1998). PCR detection based on pIPO2 revealed that this plasmid was present in 7 of 10 tested rhizosphere DNA samples, in 2 of 12 bulk soil DNA samples, but not in any of the single DNA samples from manure, seawater and wastewater (Tauch et al., 2002). Sequence comparisons made between pIPO2 and plasmid pTER331 from the soil bacterium *Collimonas fungivorans* revealed a high similarity between both plasmids (Tauch et al., 2002; Mela et al., 2008). Cryptic plasmids belonging to the pIPO2-type thus are common in the rhizosphere where they are supposed to be responsible for the mobilization and retromobilization of plasmids. In principle, the roles of these type of plasmids in horizontal gene transmission in arable plant environments have not been explored so much.

Among the species that are typical for the plant-soil ecosystem are plant pathogenic and endophytic bacteria and different plasmid types were found among species belonging to alpha, beta and gammaproteobacteria (Vivian et al., 2001). Whole-genome sequencing of the poplar endophyte *Enterobacter* sp. 638 revealed the presence of important adhesion and colonization genes on plasmid pENT638-1 (Taghavi et al., 2010), explaining the excellent colonization of strain 638 in poplar trees. This plasmid is thus of ecological relevance and supportive for the endophytic lifestyle of this strain. In *E. amylovora*, a plant pathogen commonly causing diseases in a wide variety of (rosaceous) plants, plasmids were found in different strains of this species and DNA sequences of three plasmids were annotated. Plasmid pEA29 from *E. amylovora* is a non-self-transmissible plasmid that stably replicates within its host (McGhee and Jones, 2000). On this plasmid auxiliary virulence and streptomycin resistance genes were found. DNA sequences of two other plasmids from *E. amylovora* pEU30 and pEL60, respectively, revealed strong similarities with conjugal transfer genes found in *Pseudomonas syringae* pathovar *syringae* and with pCTX-M3 plasmid of *Citrobacter freundii *(Foster et al., 2004). Plasmid pEL60 was an IncL/M type of plasmid and contained genes that are responsible for DNA repair and was proposed to increase environmental fitness of the host. The close resemblance of this plasmid with a plasmid from an animal (mouse) pathogenic species revealed that animal and plant pathogenic enteric species might share the same gene pool (Foster et al., 2004). In two strains of another plant pathogenic bacterial species, *P.* *chrysanthemi*, a gene was found that was immunologically related with the intimin-encoding gene *eae* commonly found among EHEC strains (Duarté et al., 2000). The two strains were able to kill human colon carcinoma cells, whereas a mutant defective in type 3 secretion lost the capacity to kill these cells. The combination of type 3 secretion and intimin is important for attachment to human cells and demonstrates a high conservation of virulence genes among plant and human pathogens (Duarté et al., 2000). Among strains of *Klebsiella pneumoniae* are human pathogens and (commensal) endophytes. Genome comparisons between a human pathogenic and an endophytic strain of this species revealed that there was a higher abundance of genes supposed to be responsible for survival under circumstances present inside plants like polysaccharide degradation, transport, protection against oxidative agents and nitrogen fixation (Fouts et al., 2008). In the same study, the endophytic strain was virulent in a mouse model test system, although virulence was milder in comparisons with the human pathogenic strain. *Pseudomonas aeruginosa* is a bacterial species that harbors plant as well as (opportunistic) human pathogenic strains and it was found that virulence mechanisms necessary for infection of evolutionary different hosts (vertebrate and invertebrate animal and plant species) were conserved (Rahme et al., 2000). Mutations in the genes coding for exotoxin A (*tox*A), responsible for protein synthesis inhibition in mammalian cells, phospholipase C (*plc*C), phospholipid degradation in eukaryotic cells, and *gac*A, transcriptional activator of genes responsible for production of extracellular compounds, lead to reduced virulence both in Arabidopsis and mouse test systems. Other genes responsible for multihost pathogenesis were involved in synthesis of membrane derived oligosaccharides, quorum sensing, trans-membrane export of proteins, multidrug efflux, phenazine-1-carboxylate production, motility, and surface attachment (Rahme et al., 2000). A strain of the non-pathogenic species *Wolinella succinogenes*, originating from the intestinal track system of cattle, contained many homologs of known virulence genes (Baar et al., 2003). *W. succinogenes* is a epsilon proteobacterium closely related with human pathogenic species *Campylobacter jejuni* and *Helicobacter pylori*. Next to pathogenicity gene homologs, genes proposed to be important for survival in soil and plants, including nitrogen fixation genes, were found (Baar et al., 2003). Conservation of genes that are known to be responsible for pathogenesis in humans thus are found among entirely different plant and soil bacterial species that have human pathogens as close relatives. This indicates that the plant-soil ecosystem is a source of genes that may increase fitness of many human pathogens besides *Escherichia coli* and *S. enterica* that can reside in the phytosphere.

### IN CONCLUSION

Horizontal gene transfer, either mediated by phages or by conjugal plasmids, can play a role in the acquisition of new phenotypes in human pathogens present in one of the four ecosystems relevant for arable plant production, i.e., soil, plant, animal (cattle) gut, and fresh water systems (**Figure 1**). Acquired phenotypes are related to resistance, virulence and ecological competence. All human pathogens had been prone to gene transfer events in the past and examples of recent and multiple gene transfer events were demonstrated in *Escherichia coli* serotypes O104:H4 and O157:H7. Serotype O157:H7 and some of the other O serovar types are zoonotic pathogens, which is not the case for serotype O104:H4 (entero-aggregative *Escherichia coli*), which is believed to be transmitted via humans only. From epidemiological studies it became clear that the Hamburg *Escherichia coli* O104:H4 outbreak was not transmitted via humans, but most likely via seeds. It is clear that human pathogens passing these four ecosystems might acquire mobile genetic elements, supplying them additional virulence genes and/or making them better adapted to their new environment. The combination of increased resistance to antibiotics and higher virulence levels in commensal or low-pathogenic bacteria is a major threat for human health (Szmolka and Nagy, 2013). Another hotspot boosting horizontal gene transfer thus might be, next to the mammalian gut system (Stecher et al., 2012), the rhizosphere. Studies going on from the 80-ties of the last century revealed that gene transfer via conjugation occur in the rhizosphere. New pathogenic types thus may arise at agricultural practice that are adapted to life near or inside plants; the so called phytonic types.

Products derived from plants are considered to be healthy and microbiologically safe by consumers. The public is unaware of eventual microbiological risks related to consumption of fresh produce (Beutin and Martin, 2012) and therefore emergence of phytonic human pathogens deserves attention. Appropriate and timely detection of emerging pathogens in plants and food derived from plants is of importance, but will be complicated by the flexibility of their genomes. Appearance of new features, encrypted in genomes of STEC, *S. enterica* and other human pathogenic strains, that determine fitness in plants and virulence to humans, like genes from plasmids and bacteriophage sequences, should be monitored frequently (Mellmann et al., 2011; Noguera et al., 2011a,b; Laing et al., 2012). Novel, rapid, innovative high throughput detection technologies will facilitate screenings for these regions in genomes of human pathogenic isolates from clinical and environmental samples.

## Conflict of Interest Statement

The authors declare that the research was conducted in the absence of any commercial or financial relationships that could be construed as a potential conflict of interest.
